# Physiological Aspects of Germination and Early Seedling Establishment of *Pleurotus sajor-caju* Glyceraldehyde-3-Phosphate Dehydrogenase Expressing Transgenic Rice in Saline Environment

**DOI:** 10.3389/fpls.2021.767826

**Published:** 2022-01-12

**Authors:** Zamin Shaheed Siddiqui, Gang-Seob Lee, Woosuk Cho, Mi-Jeong Jeong, Soo-Chul Park, Taek-Ryoun Kwon, Faisal Zulfiqar, Muhammad Umar, Zainul Abideen, Zaheer Uddin, Hafiza Hamna Ansari, Danish Wajid, Jung-Il Cho

**Affiliations:** ^1^Stress Physiology and Phenomic Laboratory, Department of Botany, University of Karachi, Karachi, Pakistan; ^2^Department of Agricultural Biotechnology, National Institute of Agricultural Sciences, Rural Development Administration, Jeonju, South Korea; ^3^Technology Cooperation Bureau, Rural Development Administration, Jeonju, South Korea; ^4^Department of Horticultural Sciences, Faculty of Agriculture and Environment, The Islamia University of Bahawalpur, Bahawalpur, Pakistan; ^5^Dr. Muhammad Ajmal Khan Institute of Sustainable Halophyte Utilization, University of Karachi, Karachi, Pakistan; ^6^Department of Physics, University of Karachi, Karachi, Pakistan; ^7^Crop Production and Physiology Division, National Institute of Crop Science, Rural Development Administration, Wanju, South Korea

**Keywords:** germination, *PsGPD*, amylase, chlorophyll biosynthesis, enzyme kinetics, salt stress

## Abstract

*GPD* encodes glyceraldehyde-3-phosphate dehydrogenase enzyme involved in sugar mobilization, particularly glycolysis and gluconeogenesis. The objective of this study was to determine physiological aspects of germination and early seedling establishment of *PsGPD* (*Pleurotus sajor-caju* glyceraldehyde-3-phosphate dehydrogenase) expressing transgenic rice (T5) against different salt concentrations. The T5 line that carried 2 copies of T-DNA and had the highest level of *PsGPD* expression was used in the investigation. Final germination percentage, amylase activity, reducing sugar accumulation, and chlorophyll biosynthesis were comparatively higher in *PsGPD* expressing transgenic rice against elevating saline conditions. A slow-paced conversion of porphyrin's precursors was seen through the matrix model and further elaborated by a graphical model. A sustained level of porphyrin was observed in *PsGPD* expressing transgenic rice. These data were concurrent with the relative gene expression and thermal imaging (thermography) of *PsGPD* expressing transgenic rice against salt stress. Morphological attributes also favored the salt tolerance exhibited by *PsGPD-*transformed rice.

## Graphical Abstract

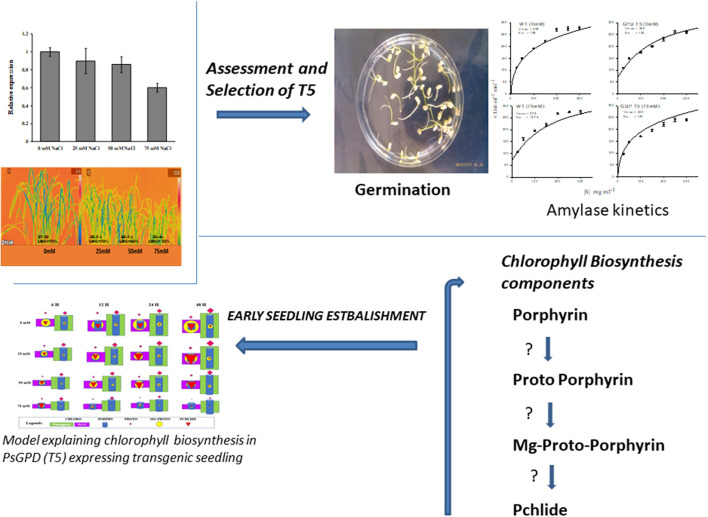

## Highlights

- PsGPD expressing transgenic rice was tested against salt stress at the most sensitive stage of plants.
- Transgenic rice (line T5) showed a proper supply of energy and substrate to complete germination.
- Seedlings were established due to rapid chlorophyll synthesis.

## Introduction

Germination and early seedling growth are critical in plant life. They are more sensitive to abiotic stress. High salt and water deficits are significant constraints that can substantially affect germination, early seedling growth, and plant productivity negatively (Siddiqui and Khan, 2011). Salt stress-induced specific cellular ion changes can alter metabolic pathways, restrict hydrolytic enzyme activity, and lead to a slow phase of seed reserve mobilization. In seeds, sugar mobilization is concurrent to amylase activity inhibited by high salt concentration in soil (Kwon et al., 2009; Siddiqui and Khan, 2010, 2011). Compromised activity of α-amylase can lead to reduced sugar translocation, which is imperative for the developing embryo. Decreased sugar content is imputable to a change in osmotic potential and decline in water uptake of growing cells (Uçarli and Gürel, 2020). Therefore, seedlings' emergence under saline condition probes the extent of seed sensitivity to salt stress (Guo et al., 2020). After germination, chlorophylls are exquisitely prerequisite complex molecules required for early seedling establishment. Chlorophyll biosynthesis is a multimeric metabolic pathway that is subjected to modulations by salt stress. Downregulation of chlorophyll biosynthesis during the greening process by salt stress is due to decreased activity of enzymes involved in the pathway. Reduced enzymatic activity is attributed to the downregulation of gene expression in salt-stressed seedlings (Siddiqui et al., 2015). Against such environmental extremes, transforming rice with salt-resistant genes effectively meets present and future food security needs. Thus, it is obligatory to identify stress-tolerant genes in seedlings (Nawaz et al., 2018).

Glyceraldehyde-3-phosphate dehydrogenase is a crucial enzyme playing a critical role in gluconeogenesis and glycolysis (Jeong et al., 2001). The *PsGPD* gene was isolated from the oyster mushroom (*Pleurotus sajor*-*caju*). It is serviceable to develop tolerant crops against various climatic crises. Yeast cells harboring the *PsGPD* gene show higher survival rate against various abiotic stresses. Potato plants transformed with the *PsGPD* gene also confer salt tolerance against salt loading (Jeong et al., 2001). However, the physiological mechanism of *PsGPD* expressing transgenic rice seed during germination and early seedling establishment against salt stress has not been highlighted so far (Jeong et al., 2000).

At present, global food security is facing a “more-consumption less-production” problem. Having feed crops with high salt tolerance that can be planted on saline soil is of great strategical importance to ensure a practical solution for food and energy production. Therefore, it would be interesting to assess the relative gene expression, germination, and early seedling performance of *PsGPD* expressing transgenic rice in a saline habitat. The objective of this study was to (1) determine *PsGPD* expressing transgenic seed germination and significant sugars mobilization with enzyme kinetic in a saline environment; and (2) investigate the biosynthetic pathway of chlorophyll supply orientation for early seedling establishment.

## Materials and Methods

### Plant Materials

Of three transgenic lines, that is, T1, T3, and T5 constitutively expressing the *PsGPD* gene under the control of a CaMV 35S promoter, the T5 line carrying two copies of T-DNA and having the highest level of *PsGPD* expression (Cho et al., 2014) was used in the experiment. Seeds of *PsGPD* expressing transgenic rice (T5) and Dongjin used in the production of transgenic lines were provided by the National Center for G.M. Crops (NCGC), National Academy of Agricultural Science (NAAS), Rural Development Administration (RDA), Korea.

### Quantitative Real-Time PCR and Thermal Sensing by FLIR SC620

Total RNAs were isolated from 15-day-old leaf samples of *GPD* transgenic rice (T5) exposed to 0, 25, 50, and 75 mM NaCl. For quantitative real-time PCR, the expression level of OsActin1 was used as an internal control to normalize real-time PCR results (Fukao et al., 2011). According to the manufacturer's instructions, all reactions were performed in triplicate using a CFX 96 Touch Deep Well Real-Time PCR System (BIO-RAD) and CFX Maestro Software (BIO-RAD). The expression level of *PsGPD* was analyzed using the comparative cycle threshold (–ΔΔCt) method. Gene-specific primers used for quantitative real-time PCR were as follows (Cho et al., 2014):

1) *PsGPD*, 5'-ATGTTCAAGTACGACTCCGTCC-3' and 5'-AGCCTTGTCTATGGTGGTGAAG-3'
2) *OsActin1*, 5'-ACAGGTATTGTGTTGGACTCTGG-3' and 5'-AGTAACCACGCTCCGTCAGG-3'

Forward-looking infrared thermal images were taken before PCR analysis. Camera lens was optimized 60 min before observation. Images were taken using approximately 640 × 480 cm rectangular wooden sheets to homogenize image backgrounds. The temperature inside the room was 35 ± 2°C and relative humidity was 65–75%. Images were taken using a FLIR SC 620 series camera having a spectral range of 7.5–13 μm, IR resolution of 640 x 480 pixels, and image frequency of 30 Hz. The camera emissivity was 0.95 with a sensitivity of 99 ± 1°C. An image temperature inspection report was generated using the FLIR software. Leaf moisture content was measured after taking thermal images according to the method of Siddiqui et al. (2014).

### Experiment for the Germination and Seedling Growth

*Pleurotus sajor-caju* glyceraldehyde-3-phosphate dehydrogenase expressing transgenic seeds were collected from NCGC. They were surface sterilized in 75% absolute alcohol for 10 min and then sterilized with 2% sodium hypochlorite for 5 min. Finally, they were washed several times with distilled water. These sterilized seeds were soaked in distilled water (control) and respective NaCl solution (25, 50, and 75%) for 10 min. Later, 20 healthy seeds from each control and treatment were transferred into 9-cm diameter Petri plates. The whole setup was kept in a Hotpack germinator. Experimental conditions were set as 25 ± 3°C with 13 h of light period (300 μmol m^−2^ s^−1^ white fluorescence light) from 06:00 a.m. to 06:30 p.m. and 20.0 ± 2°C during 11.5 h of darkness. Test solutions and distilled water were added on each alternate day. Final germination percentage and seedling lengths were recorded after 15 days. Treatment and control were replicated three times. Datasets were subjected to factorial analysis of variance in a completely randomized manner using SPSS 16.0.

### Extraction and Estimation of Total Reducing Sugar, Amylase Activities, and Enzyme Kinetics

Fifteen germinating *PsGPD* expressing seeds from each control and treatment were randomly collected and homogenized separately in chilled 100 μL Tris-HCl buffer (pH 6.6) and centrifuged at 12,000 rpm for 20 min at 2°C. Supernatants were collected to determine total reducing sugar (Nelson, 1944), amylase activity (Siddiqui and Khan, 2011), and enzyme kinetics using variable substrate concentrations. For amylase activity, 1,000 μL extract was reacted with a 1,000 μL substrate (starch). After 20 min, the reaction was stopped using I N NaOH, and the reaction mixture was treated with 200 μL dinitro salicylic acid. Optical density was read at 540 nm, and maltose released as an enzyme product was estimated using a standard curve. For enzyme kinetic (substrate saturation kinetic), 0.5 to 3.0 g ml^−1^ substrate was used. Substrate saturation kinetic curve was made using software GRAPHAD PRISM 5.0.

### Experiment for Chlorophyll Biosynthesis Assessment

*Pleurotus sajor-caju* glyceraldehyde-3-phosphate dehydrogenase seeds were surface-sterilized similarly as described in Experiment 1. Sterilized seeds were soaked in water, incubated in the dark at 26°C for 2 h, and transferred to three 40-cm diameter pots (triplicate) kept at 25 ± 3°C. In each pot, more than 250 seedlings were grown. Forty-eight etiolated seedlings shared in a hydroponic tank with freshly prepared one-tenth Hoagland culture solution. Four tanks of each containing 0, 25, 50, or 75 mM were used, with the 0% serving as control. Photoperiod, light intensity, and relative humidity were 13 h, 300 μmol m^−2^ s^−1^, and 70%, respectively. Twenty-five seedlings from each treatment and control were collected randomly after 6, 12, 24, and 48 h of treatment. All treatments and control were replicated four times and subjected to ANOVA using SPSS 16.0.

### Chlorophyll Determination

Fifty mg were extracted with 1,000 μL of ethanol (95%), homogenized, and centrifuged at 10,000 rpm for 10 min. The absorbance of the solution was measured at 653 and 666 nm with a spectrophotometer (Shimadzu, Japan). Total chlorophyll pigments were calculated using the equation of Lichtenthaler and Wellburn (1985):

Chl a = 15.65 A_666_ – 7.340 A_653_,

Chl b = 27.05 A_653_ – 11.21 A_666_,

Total chlorophyll = Chl a + Chl b.

### Porphyrin Determination

To determine porphyrin precursors for chlorophyll biosynthesis, that is, magnesium protoporphyrin (Mg-proto), protoporphyrin IX (Proto), and protochlorophyllide (Pchlide), leaves from each treatment and control were extracted with a solvent mixture of 100 μL acetone and 0.1 N NaOH at a ratio of 9:1 (v/v). Later, the homogenate was centrifuged at 12,000 rpm for 15 min. The remaining pellet was extracted with a solvent mixture of 100 μL acetone and NH_4_OH at a ratio of 8:2 (v/v). The pooled supernatant was extracted with 250 μL petroleum ether for protochlorophyllide, whereas 1,300 μL saturated NaCl and 40 μL 0.5 M dihydrogen potassium phosphate (KH_2_PO_4_) were added into the acetone extract solution for Mg-proto. Similarly, a mixture of 1,000 μL ethyl acetate and acetic acid at 6:2 (v/v) was used to extract protoporphyrin IX. These three porphyrin precursors were then quantified with the method of Kahn et al. (1976). However, the mole percent of porphyrin was calculated in the following manner:


$$\begin{array}{l} \text{Porphyrin }\left(\%\right)\;=\;\left[\frac{Proto\;\left(or\;MgProto\;or\;Pchlide\right)}{\left(Proto\;+\;MgProto\;+\;Pchlide\right)}\right]\;\times \;100 \end{array}$$

## Results

*Pleurotus sajor-caju* glyceraldehyde-3-phosphate dehydrogenase expressing transgenic rice concerning germination and early seedling responses against saline habitat were studied. Carbohydrate metabolism was recorded using amylase activity, enzymes–substrate reaction velocity, enzyme–substrate affinity, and reducing sugar mobilization. *PsGPD* expressing transgenic rice (T5) produced substantial final percent germination compared with wild type (WT) in salt stress (Table 1). The final percent germination of a transgenic line (−10 to −26%) was much better than the WT (−5.0 to −36%) at 75 mM NaCl. It was noticed that *PsGPD* expressing transgenic line showed better germination than the WT under salt stress. Under salt stress, root growth was severely affected as compared to shoot growth (Table 1). It was found that root and shoot growths of transgenic ones were less affected than those of WT.

**Table 1:** Final percent germination and early seedling growth of PsGPD expressing transgenic rice in saline environment.

**Treatments**	**Germination (%)**	**Root Length (cm)**	**Shoot Length (cm)**	**R-S Ratio**
WT (0 mM)	95 ± 1.2^a^	3.2 ± 0.06^a^	6.90 ± 0.054^a^	0.46
GPD (0 mM)	95 ± 2.5^a^	3.4 ± 0.06^a^	7.20 ± 0.064^a^	0.47
WT (25 mM)	90 ± 1.05^a(−5.0%)^	2.95± 0.04^a(−7%)^	6.4 ± 0.033^b(−7.2%)^	0.46
GPD (25 mM)	90 ± 1.20^a(−5.0%)^	3.05± 0.04^a(−10%)^	6.8 ± 0.044^b(−5.5%)^	0.44
WT (50 mM)	80 ± 1.20^b(−15%)^	2.50± 0.02^b(−21%)^	5.6 ± 0.024^c(−18.8%)^	0.44
GPD (50 mM)	85 ± 1.75^b(−10%)^	2.75± 0.02^b(−19%)^	6.4 ± 0.054^c(−11.1%)^	0.42
WT (75 mM)	60 ±1.05^c(−36%)^	1.20 ± 0.04^c(−62.5%)^	3.2 ± 0.02^d(−53.6%)^	0.37
GPD (75 mM)	70 ±1.25^c(−26%)^	2.65 ± 0.07^c(−22.05%)^	5.8 ± 0.08^d(−19.4%)^	0.45

*(±) Mean standard error and similar alphabets are not significantly different at p < 0.05 (Bonferroni). Values in parenthesis expressed percent differences compared with control*.

**Figure 1:**
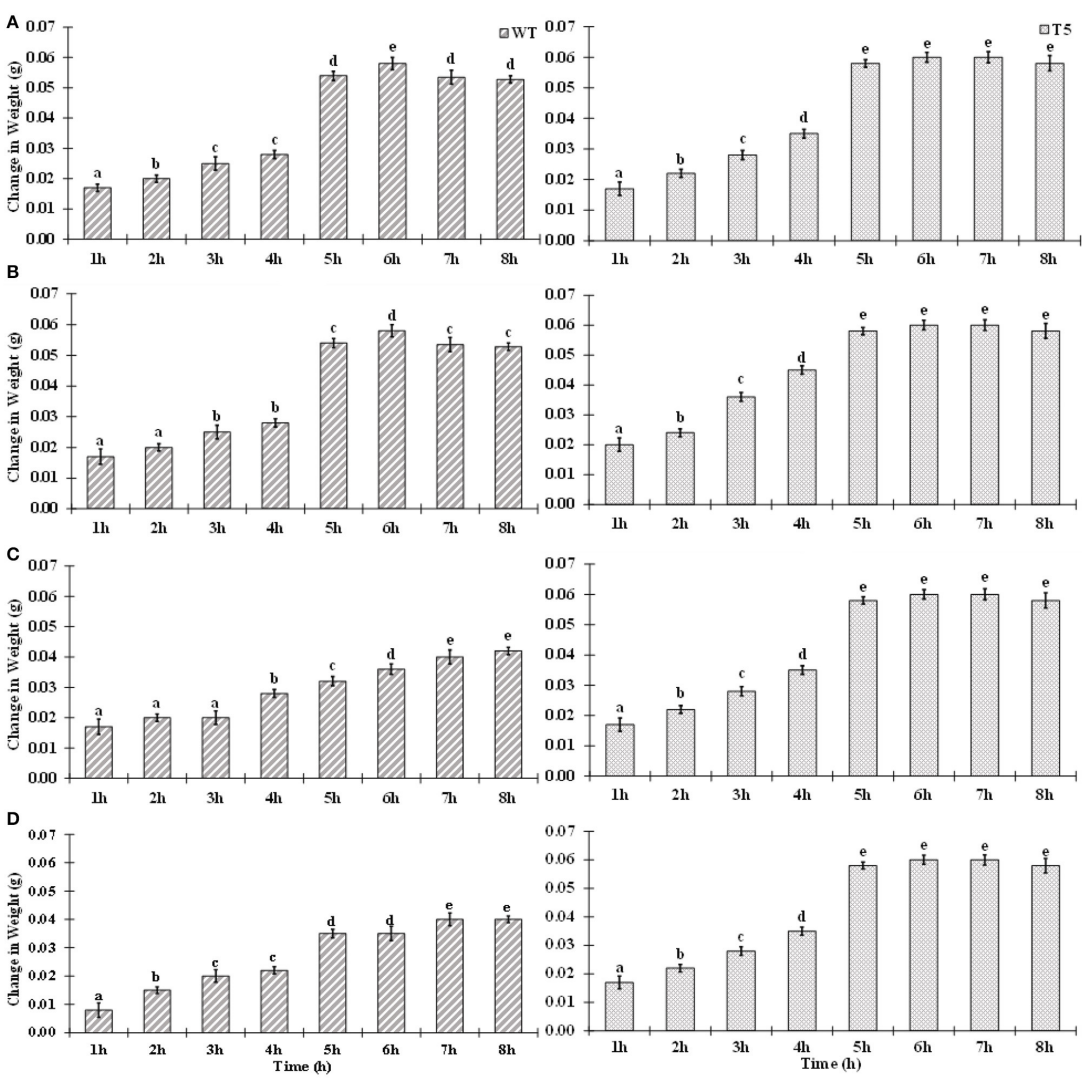
Water uptake of WT and PsGPD transgenic rice (T5) during germination in saline environment. Symbol stand for [a] = 0 mM, [b] = 25 mM, [c] = 50 mM & [d] = 75 mM. Vertical lines on each bar represent (±) mean standard error. Similar alphabets are not significantly different at p < 0.05 (Bonferroni).

Wild type showed more significant decrease in root growth (−62.05%) than the transgenic line (−22%) under 75 mM salt stress over a non-saline control. It was observed that shoot growth in T5 was less affected than that in WT. A drastic decrease was noticed in shoot length of WT (−53%) than in T5 (−19%). A gradual reduction in seedling growth was noted in salt-treated *PsGPD* expressing rice as compared to the control. Root-to-shoot ratio was not significantly varied for salt stress. Change in weight due to water uptake was increased with an increase in time. However, after the 7th h, water uptake was non-significantly decreased. Compared with the WT, water uptake in *PsGPD* expressing transgenic rice was significantly enhanced by 25, 50, or 75 mM salt stress (Figure 1).

**Figure 2:**
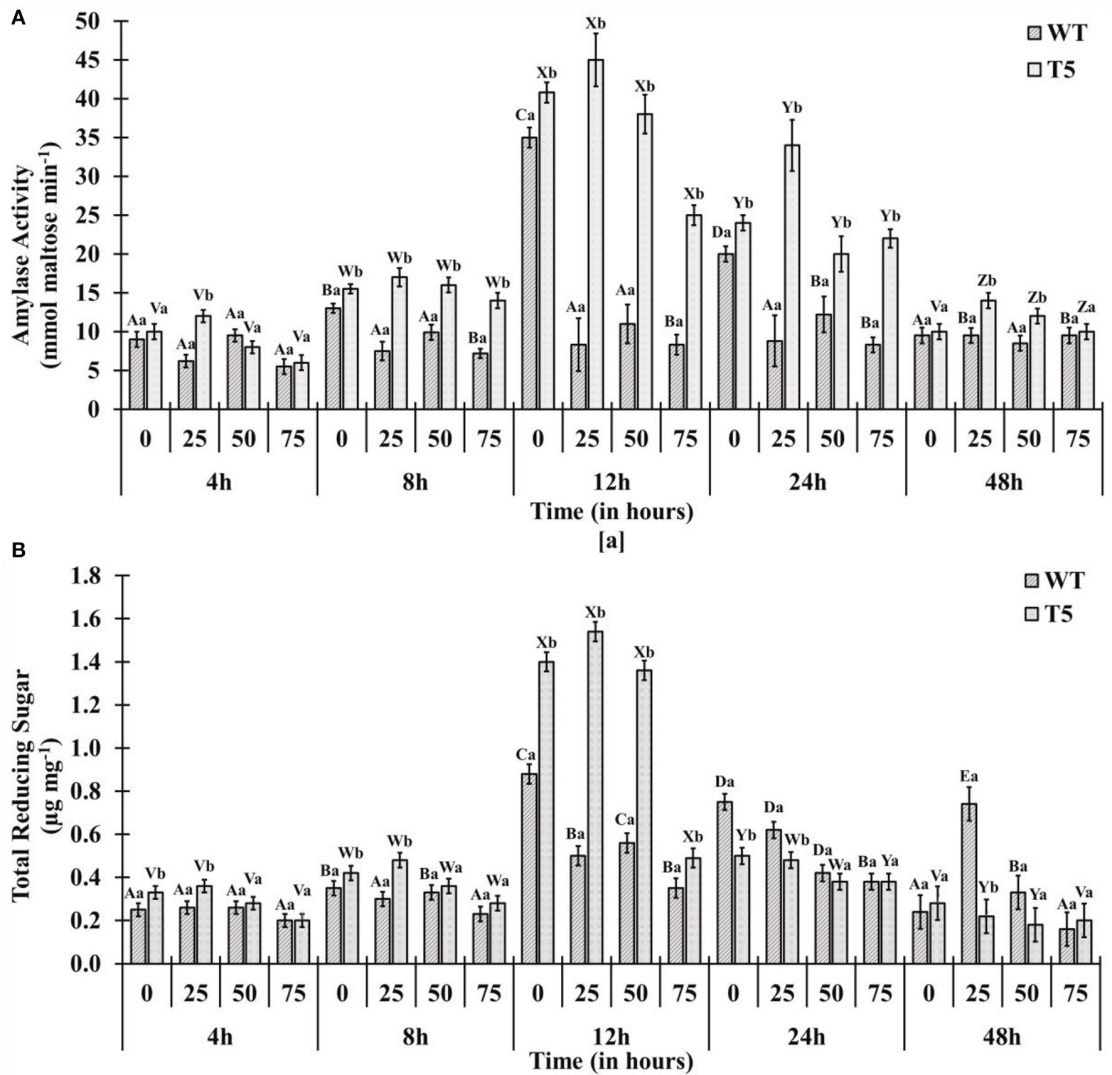
**(A)** Amylase activity and **(B)** total reducing sugars of PsGPD expressing transgenic rice (T5) in saline environment (0, 25, 50, and 75 mM NaCl). Vertical lines on each bar represent (±) mean standard error. Similar *small* alphabets are not significantly different at *p* < 0.05 among WT and T-5 whereas similar capital alphabets showed non-significant difference with time (Bonferroni).

**Figure 3:**
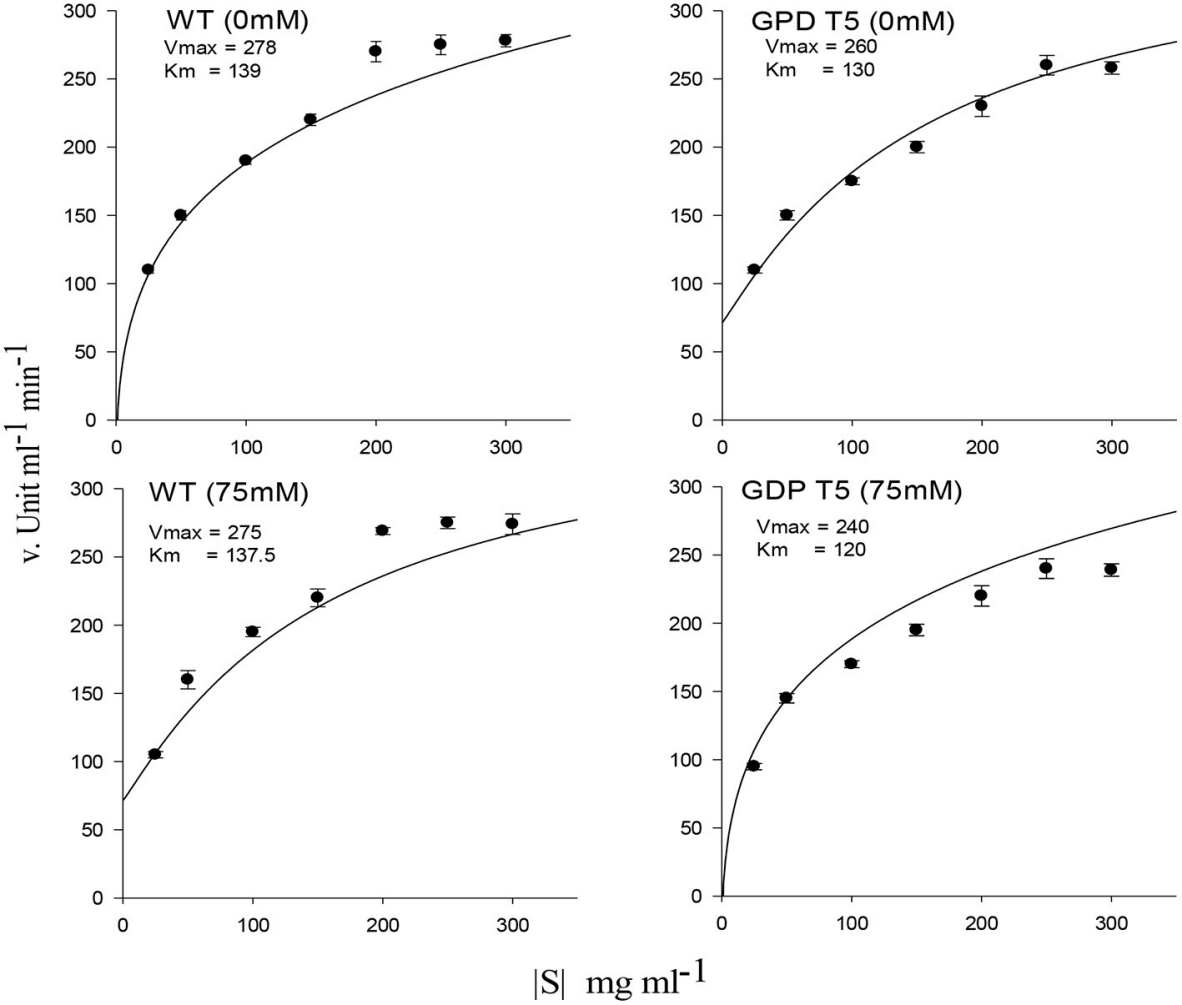
Amylase enzyme kinetics showing maximum enzymes velocity (V_max_) and affinity (K_m_) of PsGPD expressing transgenic rice (T5) in saline environment (75 mM NaCl). Vertical lines represent (±) mean standard error.

Amylase activity and mobilization of reducing sugar (total reducing sugar content) were reached to maximal in early hours under 25 and 50 mM salt stress compared with 75 mM salt stress, displaying quick mobilization of stored sugar. It was noted that amylase activity and reducing sugar were optimum at 12 h under 25 and 50 mM salt stress, whereas T5 under 75 mM NaCl expressed the highest reduction in sugar and amylase at 12 h (Figure 2). Enzyme's reaction velocity (V_max_) and substrate affinity (K_m_) were measured using variable substrate concentrations. It was comparatively less in 75 mM treated samples than in control and other treatments. Amylase activity of sugar mobilization after 48 h was the highest under 25 mM salt stress and the slowest under 75 mM salt stress among all treatments, including control and other salt stress treatments (Figure 3). It was observed that sugar mobilization was quicker in T5 line than in WT due to substantially high amylase activity under salt stress. Moreover, T5 line showed better and faster amylase activity and sugar mobilization than WT.

**Figure 4:**
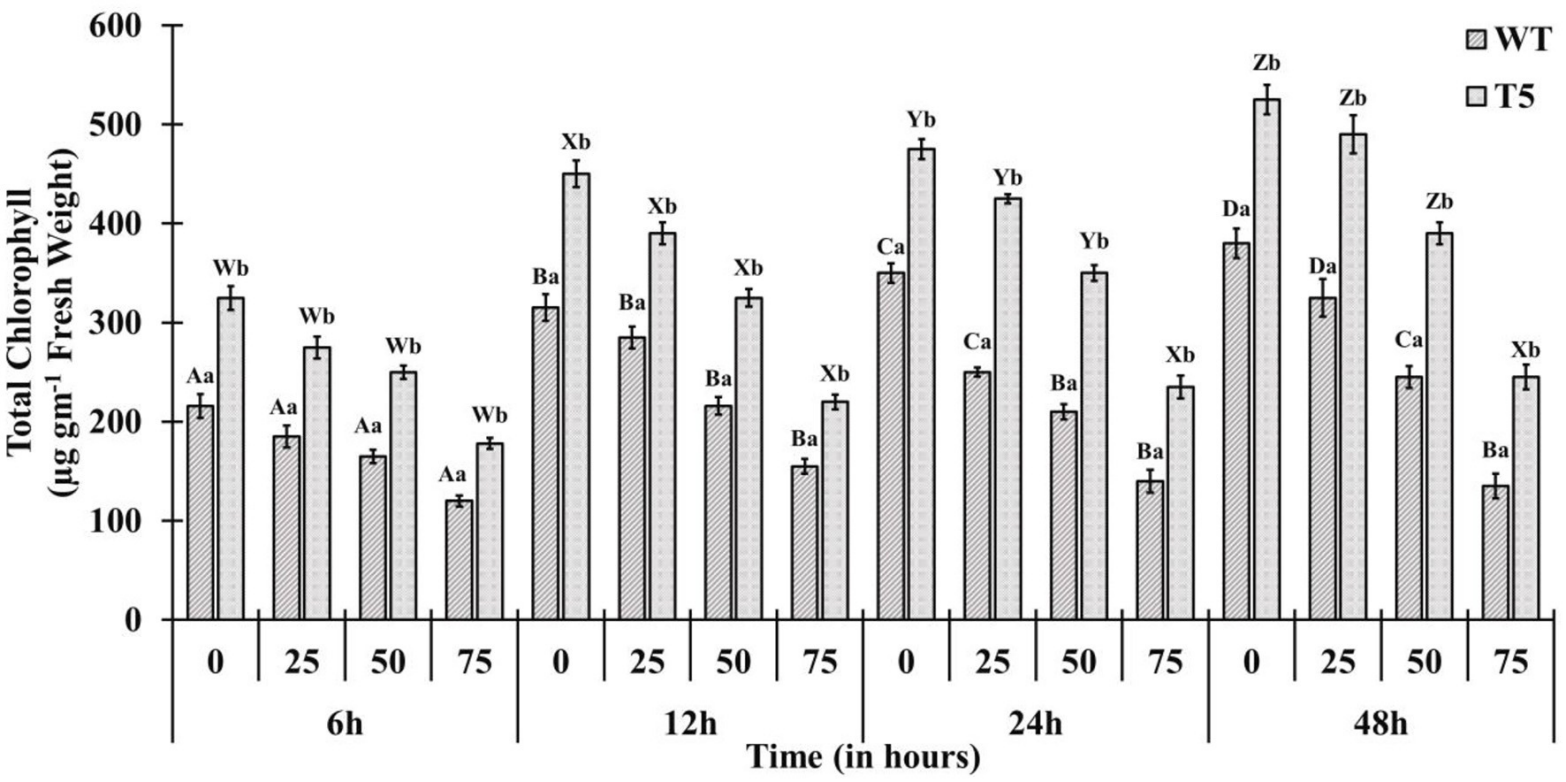
Total chlorophyll accumulation in *PsGPD* expressing transgenic rice (T5) in saline habitat (0, 25, 50, and 75 mM NaCl). Vertical lines on each bar represent (±) mean standard error. Similar *small* alphabets are not significantly different at *p* < 0.05 among WT and T-5 whereas similar capital alphabets showed non-significant difference with time (Bonferroni).

**Figure 5:**
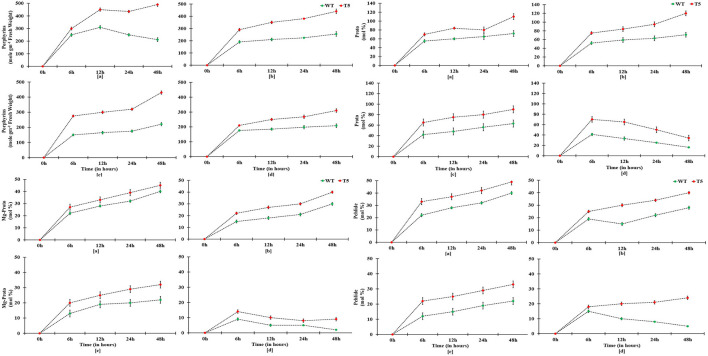
Porphyrins and porphyrin precursors synthesis in PsGPD expressing transgenic rice (T5) under saline environment. Alphabets a, b, c, and d on the horizontal axis represents the salinity levels 0, 25, 50, and 75 mM NaCl, respectively. Vertical lines represent (±) mean standard error.

Chlorophyll accumulation in *PsGPD* expressing transgenic seedlings was significantly inhibited by 75 mM salt stress. However, salt stress at 25 and 50 mM showed non-significant inhibition (Figure 4). In a saline medium, whole porphyrins precursors (porphyrin, Mg-proto, and Pchlide) and porphyrin could be detected within 6 h after each treatment. Their synthesis was slower under 75 mM salt stress than that under 25 or 50 mM salt stress (Figure 5). We observed that the mole percentage of porphyrins increased with increasing concentration of salt and exposure duration. However, the concentration of individual porphyrin (mole percentage) in WT was somewhat similar to that in transgenic lines. Two distinct phases were observed in the mole percentage of total porphyrins and its precursor. The first phase was fast, occurring within 6 h. The second phase was slightly slower and somewhat steady state. Salt stress significantly affected the accumulation of porphyrins precursors. However, chlorophyll biosynthesis in *PsGPD* expressing transgenic seedlings was less affected by 25 or 50 mM NaCl than by 75 mM NaCl. A significant positive correlation was observed among porphyrins and chlorophyll in PsGPD expressing transgenic rice (*r* = 0.95) in comparison with that in WT (*r* = 0.62) (Table 2).

**Table 2:** Pearson's correlation between Chlorophyll biosynthesis and its precursors in WT and PsGPD expressing transgenic rice (T5) in saline environment.

**WT**	**Porphyrin**	**Proto**	**Mg-proto**	**Pchlide**	**Chlorophyll**
Porphyrin	1				
Proto	0.46^ns^	1			
Mg-proto	0.54*	0.92**	1		
Pchlide	0.56*	0.88**	0.97**	1	
Chlorophyll	0.62*	0.86**	0.94**	0.90**	1
T5					
Porphyrin	1				
Proto	0.71*	1			
Mg-proto	0.87**	0.87**	1		
Pchlide	0.94**	0.74*	0.93**	1	
Chlorophyll	0.95**	0.81**	0.94**	0.96**	1

* *Represents significant at p < 0.05*,

** *represents significant at p < 0.01 and ns represents non-significant*.

**Figure 6:**
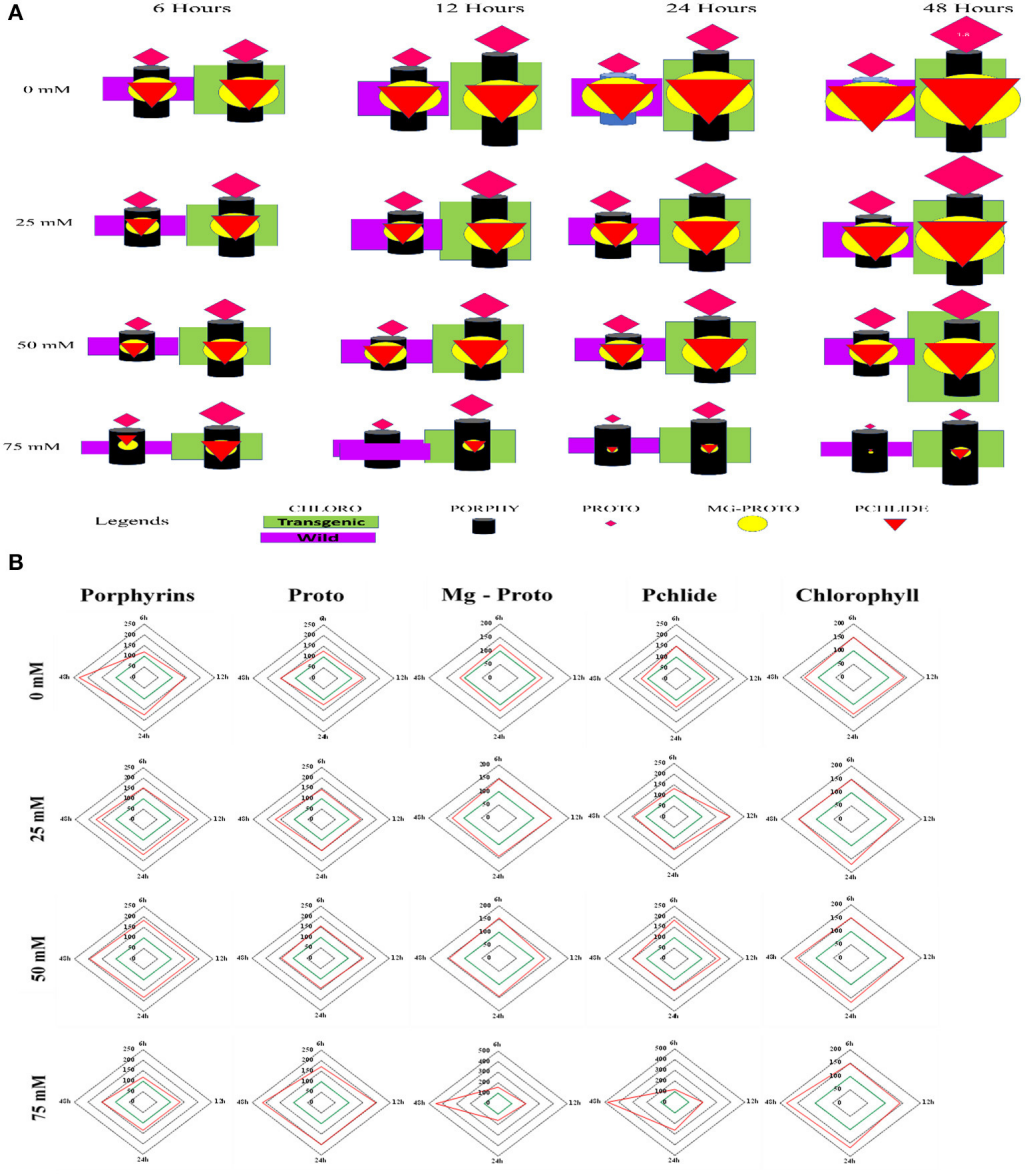
**(A)** Represents the matrix model of chlorophyll content (unit), porphyrins (unit), protoporphyrin (unit), Mg-protoporphyrin (unit), and Pchlide (unit) concentrations in the wild type (purple rectangle) and transgenic lines (green rectangle). The cylinder shape presents porphyrin concentration, diamond shape presents protoporphyrin concentration, circle presents Mg-protoporphyrin concentration, and triangle presents Pchlide concentration. **(B)** Represents the percentage increase of chlorophyll biosynthesis and its precursors in PsGPD expressing transgenic rice (T5) against the WT in saline environment.

Figure 6A shows a matrix model to describe variations in chlorophyll biosynthesis between the WT and the transgenic line (T5). There were 16 different matrix elements for combining timeslots (6, 12, 24, and 48 h) and salinization (0, 25, 50, and 75 mM). Each matrix element had two parts based on WT and T5. WT was shown on the left-hand side and T5 was shown on the right-hand side. Each matrix element had five geometrical shapes: rectangle, cylinder, diamond, circle, and triangle representing chlorophyll, porphyrin, proto, Mg-proto, and Pchlide, respectively. These rectangles had the same length but varying width depending on the value of chlorophyll. Both rectangles (purple and green) (i.e., chlorophyll) showed increases in size with increasing time under a non-saline condition. The same was true under salinity stress condition of 25 mM. For salinity stress conditions at 50 and 75 mM, the color first increased and then decreased (sizes of rectangles first rose, then dropped). The cylindrical shape (i.e., porphyrins) increased monotonically for stress conditions having a salinity of 25, 50, or 75 mM. Sizes of diamonds representing proto were increased monotonically in non-saline condition and under 25 or 50 mM salinity stress condition. The situation reversed for 75 mM salinity stress condition. The diamond (proto) size decreased monotonically. The variation in the size of circle (Mg-proto) followed the same pattern as that of the diamond. Triangular shapes representing Pchlide increased with time for non-saline condition and salt stress conditions except for 75 mM salinity stress, which decreased monotonically.

Figure 6B presents the percentage in chlorophyll content and its precursors of *PsGPD* expressing transgenic lines (T5) against WT (considered 100%). Under non-saline conditions, the transgenic line (T5) expressed the highest percentage increase in porphyrins (≈ 250%) in comparison with the WT at 48 h. At 75 mM salt stress, the percentage increase of porphyrin was the least whereas the percentage increase of proto was highest in PsGPD expressing transgenic rice. Both Mg-proto and Pchlide of T5 displayed an abrupt rise (≈ 450%) at 48 h under 75 mM salt stress. Chlorophyll content was higher in PsGPD expressing transgenic rice than in the WT against 75 mM salt stress at 48 h.

**Figure 7:**
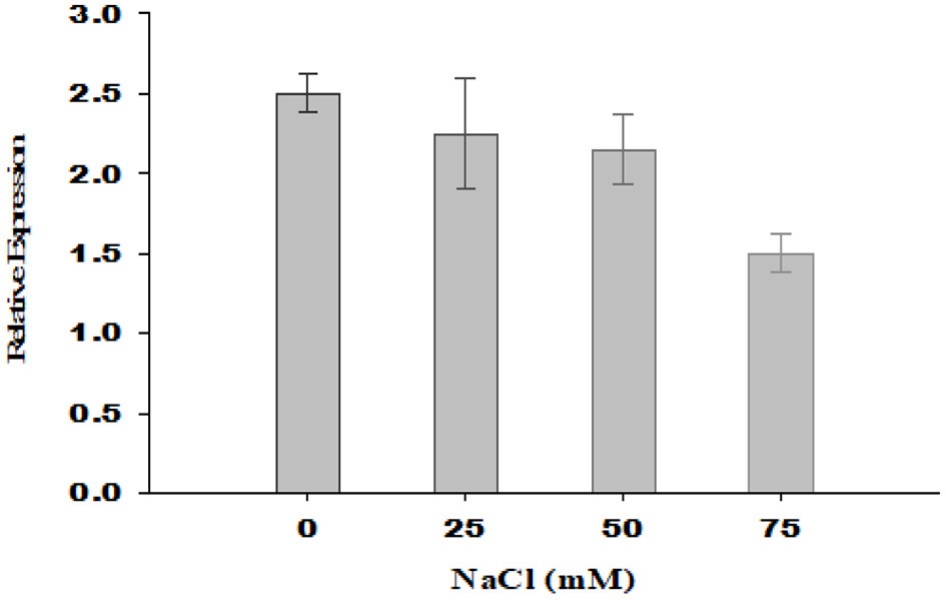
Relative gene expression of *PsGPD* expressing transgenic rice in salt stress environment. The expression level of PsGPD gene in seedlings grown under non-saline condition was arbitrarily considered as 1. Vertical lines on the bar graph showed (±) mean standard error.

**Figure 8:**
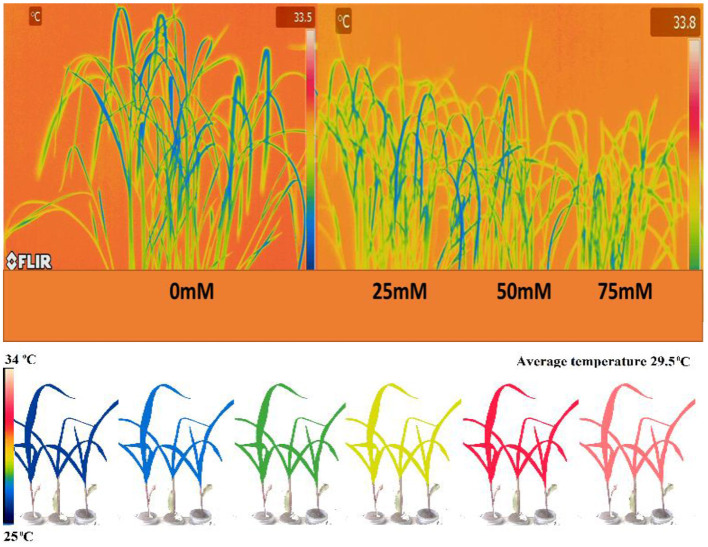
Thermal images were taken by FLIR camera model SC620. An autogenerated model for the comparison of temperature and moisture content of the *PsGPD* expressing transgenic rice (T5) is also presented (Siddiqui et al., 2015). LMC, leaf moisture content.

With an increase in salt stress, the relative expression of PsGPD gene in T5 rice was decreased, which correlated with water uptake, chlorophyll content, porphyrin, and its precursors, and enzymatic (amylase) activity results (Figure 7). Thermal images obtained from infrared camera signified the plant's water status, showing that a gradual increase in leaf temperature might be due to less water in salt-affected leaf in T5 transgenic line under various salt concentrations (Figure 8). A leaf model showed different colors that representing changes in leaf temperature on camera screen. In summary, the T5 line showed better water uptake, chlorophyll, and precursor synthesis than the WT under salt stress.

## Discussion

Adaptation of plants to salinity during germination and the early seedling stage is pivotal for the establishment of plants (Kaymakanova, 2009; Siddiqui and Khan, 2010). Most transgenic lines that exhibit salt tolerance genes have been tested during plant growth stage. However, plants during germination and early seedling are more sensitive to abiotic stress than those during growth (Cho et al., 2014; Siddiqui et al., 2015). In this study, *PsGPD* expressing transgenic rice (T5) produced a substantially better final percent germination and seedling growth than WT in a salt stress environment. We inferred that *PsGPD* expressing transgenic rice showed improved germination due to high transcriptional induction of hydrolytic enzyme (α-amylase) responsible for sugar mobilization in the developing embryo. Optimum sugar translocation provides energy, maintains osmotic potential, and increases water uptake for the developing radicle (Debeaujon et al., 2000; Fengshan et al., 2004; Siddiqui and Khan, 2010), which was attributable to an increase of germination percentage in *PsGPD* expressing transgenic rice (70%) in comparison with that in WT (60%) against 75 mM salt stress. Increased root length of *PsGPD* transgenic rice (-22.05%) in comparison with the WT (−62.5%) against 75 mM salt stress indicated improved root morphological plasticity (Siddiqui and Khan, 2010). The key to coping with salt stress is the plasticity of the root architecture and expansion system, which preclude salt accumulation in the root and facilitate continuous water uptake from a saline soil (John et al., 2008). This was concurrent with our data of water uptake, which showed a significant increase in *PsGPD* expressing transgenic rice compared with WT against salt stress (Figure 1). Increased amylase activity in *PsGPD* expressing transgenic rice due to the high transcriptional induction was attributed to tolerant genes against salt stress. This increased amylase activity resulted in elevated level of total reducing sugar in *PsGPD* expressing transgenic rice to support cell homeostasis and mitigate the risk of cell shrinkage and loss of turgidity (Cho et al., 2014). Increased amylase activity explicitly reflects data of increased reaction velocity and substrate affinity in *PsGPD* expressing transgenic rice (Figure 3).

After germination, as the growth of young seedlings proceeds, chlorophyll biosynthesis is the primary and mandatory physiological mechanism which can be significantly inhibited by a saline environment (Kwon et al., 2009; Cho et al., 2014; Siddiqui et al., 2015). *PsGPD* expressing transgenic rice responded to salt stress by less drastically scavenging porphyrin intermediates, resulting in increased chlorophyll content compared with WT against salt stress (Figure 4). The sustained level of porphyrin intermediates supported this improved biosynthesis. Their synchronized conversion against salt stress coincided with their increased salt tolerance in *PsGPD* expressing transgenic rice (Figure 5). The compromised chlorophyll content and slow pace conversion of porphyrin precursors at higher salinity (75 mM) were characterized by an enzymatic level inhibition due to salt stress. Enzymes such as Mg-protoporphyrin IX mono-methyl ester oxidative cyclase, Mg-Proto-methyl-esterase, and Mg-2, 4-divinyl-protoporphyrin reductase are involved in the chlorophyll biosynthetic pathway (Siddiqui, 2007). The correlation of chlorophyll with porphyrin was significantly higher in PsGPD expressing transgenic rice (*r* = 0.95) than in the WT (*r* = 0.62) (Table 2). This also demonstrated the lesser accumulation and more degradation of photosensitizing porphyrin precursors in *PsGPD* expressing transgenic rice to reduce the oxidative stress caused by salt stress.

A matrix model was developed to observe the significant difference in chlorophyll content, porphyrins, and its precursors between the WT and *PsGPD* expressing transgenic rice. Figure 5 presents 16 different matrix elements for a combination of timeslots (6, 12, 24, and 48 h) and salt stress (0, 25, 50, and 75 mM). Each matrix element consists of two parts. The left-side part represents characteristics of the WT, and the right-side part represents characteristics of *PsGPD* expressing transgenic rice. Each part has five different geometrical shapes representing chlorophyll content (rectangle), porphyrin (cylinder), protoporphyrin (diamond), Mg-protoporphyrin (circle), and Pchlide (triangle). Under an unstressed environment, the chlorophyll content increased in the WT and *PsGPD* expressing transgenic rice. From the data and the model (Figure 5), it was evident that *PsGPD* expressing transgenic line had relatively higher chlorophyll content under both unstressed and stressed conditions than the WT. The conversion of porphyrin precursors at Mg-proto and Pchlide levels was smoother in *PsGPD* transgenic lines, which caused considerably higher chlorophyll content against elevated levels of salt stress.

The decrease in chlorophyll content, porphyrins, and its precursors indicated that higher salt levels inhibited the *de novo* synthesis of chlorophyll and its precursors (Siddiqui et al., 2021). *PsGPD* expressing transgenic rice showed extremely low porphyrin precursors at 48 h, depicting the maximum conversion of precursors into porphyrin to generate chlorophyll molecules. The inhibition of precursor conversion at Mg-proto and Pchlide level and *de novo* synthesis was dominant in the WT and comparatively improved in *PsGPD* transgenic rice. The porphyrin was increased monotonically in both unstressed and stressed conditions (0, 25, 50, and 75 mM), which explained that salt stress inhibition majorly affected the conversion of proto to Mg-proto and Mg-proto to Pchlide. Activities of enzymes such as Mg-protoporphyrin IX mono-methyl ester oxidative cyclase, Mg-proto-methyl-esterase, and Mg-2, 4-divinyl-protoporphyrin reductase were affected by salt stress which might have caused such hindrance or slower pace conversion of precursors (Siddiqui, 2007). Elevated porphyrin (≈ 250%) level in *PsGPD* expressing transgenic rice might be due to the smoother conversion of its precursors against salt stress. Lesser enzymatic inhibition during biosynthesis was favored by tolerance and greater efficiency of substrate availability for enzymes. The abrupt increase of Mg-proto and Pchlide justified the availability of substrate for enzymes to biosynthesize an optimum mole percent of porphyrin, which might have led to a sustained chlorophyll level in transgenic plants against salt stress. This was also evident by the significant positive correlation observed in transgenic rice (*r* = 0.95) in comparison with the WT (*r* = 0.62) (Table 2). Although the correlation of Mg-proto and Pchlide in transgenic rice (*r* = 0.94 and *r* = 0.96) was similar to that in the WT (*r* = 0.97 and *r* = 0.90), a significant difference occurred at the porphyrin level. This explained the slow or inhibited conversion of Mg-proto and Pchlide levels to form porphyrin.

Thermal images (thermography) of *PsGPD* expressing transgenic rice showed a lesser increase in leaf temperature against salt stress (Figure 8). This might be due to balanced water uptake from the saline soil as leaf temperature is directly related to the leaf's rate of evaporation and transpiration. The cooling effect of transpiration is used to maintain leaf temperature rises due to stomatal closure in times of water stress (Siddiqui et al., 2014). At 75 mM salt stress, the leaf temperature was relatively higher, coinciding with the downregulation of the *PsGPD* gene observed in relative gene expression results (Figure 7).

Conclusively, this study witnessed salt tolerance conferred by the *PsGPD* gene as its downregulation caused a decrease in water uptake, resulting in lower hydrolytic enzyme activity, germination rate, retarded morphological attributes, and slow pace chlorophyll biosynthesis with an increase in leaf temperature. The matrix model, graphical representation of chlorophyll and porphyrin precursors and thermal imaging through infrared radiation, exemplified the physiological mechanism and perturbations caused by salt stress. These perturbations were more dominant at higher salt stress (75 mM) which depicted that salt tolerance of *PsGPD* gene to lower salt concentration was more than WT during early seedling establishment.

## Data Availability Statement

The raw data supporting the conclusions of this article will be made available by the authors, without undue reservation.

## Author Contributions

ZS designed the experimental work. ZU developed a matrix model. All the authors are equally contributed to this manuscript and reviewed the draft.

## Conflict of Interest

The authors declare that the research was conducted in the absence of any commercial or financial relationships that could be construed as a potential conflict of interest.

## Publisher's Note

All claims expressed in this article are solely those of the authors and do not necessarily represent those of their affiliated organizations, or those of the publisher, the editors and the reviewers. Any product that may be evaluated in this article, or claim that may be made by its manufacturer, is not guaranteed or endorsed by the publisher.
